# Herpes simplex virus UL56 interacts with and regulates the Nedd4-family ubiquitin ligase Itch

**DOI:** 10.1186/1743-422X-7-179

**Published:** 2010-08-03

**Authors:** Yoko Ushijima, Chenhong Luo, Maki Kamakura, Fumi Goshima, Hiroshi Kimura, Yukihiro Nishiyama

**Affiliations:** 1 Department of Virology, Nagoya University Graduate School of Medicine, 65 Tsurumai-cho, Showa-ku, Nagoya 466-8550, Japan

## Abstract

**Background:**

Herpes simplex virus type 2 (HSV-2) is one of many viruses that exploits and modifies the cellular ubiquitin system. HSV-2 expresses the tegument protein UL56 that has been implicated in cytoplasmic transport and/or release of virions, and is a putative regulatory protein of Nedd4 ubiquitin ligase. In order to elucidate the biological function of UL56, this study examined the interaction of UL56 with the Nedd4-family ubiquitin ligase Itch and its role in the regulation of Itch. Additionally, we assessed the similarity between UL56 and regulatory proteins of Itch and Nedd4, Nedd4-family-interactins proteins (Ndfip).

**Results:**

UL56 interacted with Itch, independent of additional viral proteins, and mediated more striking degradation of Itch, compared to Nedd4. Moreover, it was suggested that the lysosome pathway as well as the proteasome pathway was involved in the degradation of Itch. Other HSV-2 proteins with PY motifs, such as VP5 and VP16, did not mediate the degradation of endogenous Itch. Ndfip1 and Ndfip2 were similar in subcellular distribution patterns to UL56 and colocalized with UL56 in co-transfected cells.

**Conclusions:**

We believe that this is the first report demonstrating the interaction of a HSV-specific protein and Itch. Thus, UL56 could function as a regulatory protein of Itch. The mechanism, function and significance of regulating Itch in HSV-2 infection remain unclear and warrant further investigation.

## Background

Viruses act as intracellular parasites, depending heavily on functions provided by their host cells, and have evolved diverse strategies to exploit the biology and biochemistry of hosts for their benefit [1]. The ubiquitin system is one of the mechanisms exploited by many viruses; it is involved in viral assembly and release, viral transcriptional regulation, viral immune invasion, and the suppression of apoptosis [2,3]. The ubiquitin system is a key regulatory mechanism for a diversity of cellular processes including protein turnover, protein sorting and trafficking, signal transduction, and cell-cycle control [4]. Ubiquitination is executed by a hierarchical cascade of enzymes [5]. E3 ubiquitin ligases act as major specificity determinants of the ubiquitin system by facilitating the transfer of ubiquitin to lysine residues of the target proteins. The human genome encodes more than 600 putative E3 ligases [6], which generate the diversity in the ubiquitin system. E3 ligases are classified into two main groups: really interesting novel genes (RING) and homologous to E6AP carboxyl terminus (HECT) proteins. The neuronal precursor cell-expressed developmentally down-regulated 4 (Nedd4) family, comprised of nine members, is one of the main HECT E3 protein families.

Viruses encode their own E3 ligases, de-ubiquitinating enzymes (DUBs) and adaptor/regulatory proteins to modify the host's ubiquitin system [2,3]. Herpes simplex virus (HSV) is a large, enveloped, double-stranded-DNA virus, which can cause various mild and life-threatening diseases, including herpes labialis, genital herpes, keratitis, encephalitis, and neonatal herpes [7]. HSV encodes a ubiquitin ligase (ICP0) [8,9] and a DUB (UL36) [10]. In addition, we identified that the HSV type 2 (HSV-2) tegument protein UL56 is a putative regulatory protein of Nedd4 E3 ligase [11], specifically involved in protein stability and subcellular localization. UL56 induces phosphorylation of Nedd4 and promotes the proteasome-mediated degradation by increasing ubiquitination of Nedd4, however UL56 itself is not ubiquitinated [11]. UL56 relocates Nedd4 primarily to the trans-Golgi network (TGN) and partially to endosomes [12].

Approximately half of the 74 genes encoded by HSV are accessory genes that are not essential for viral replication in cell-culture system [7,13,14]. *UL56 *gene is an accessory gene encoded by most members of the Alphaherpesvirinae family (References are listed in [12]). Interestingly, UL56-deficient HSV-1 is substantially less neuroinvasive in vivo [15,16], although little is known about the molecular mechanisms of the attenuation. Previously, we have shown that UL56 deficiency reduces the titer of extracellular HSV-2 [12]. These data suggest that UL56 facilitates the cytoplasmic transport of virions from the TGN to the plasma membrane and/or the release of virions. In addition, we found that UL56 interacts with two other proteins: KIF1A [17], the neuron-specific kinesin; and HSV-2 UL11 [18], a tegument protein that has dynamic membrane-trafficking properties [19] and plays a role in the envelopment and egress of viral nucleocapsids [20]. These interactions also support the view that UL56 is involved in transports of vesicles and virions, however the precise roles and functions of UL56 remain elusive.

**Figure 1:**
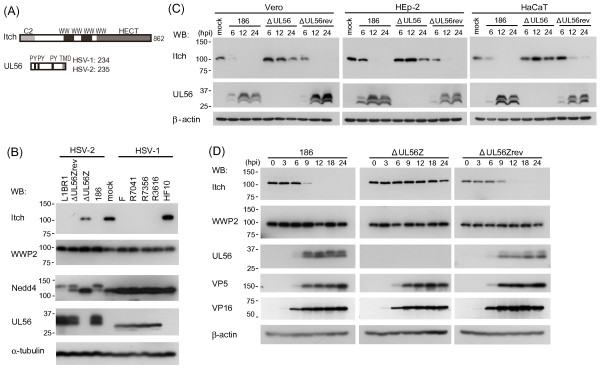
**HSV infection causes a marked decrease of Itch in the presence of UL56**. (A) Schematic representation of Itch and UL56. Itch (862 aa) contains a Ca^2+^/lipid binding C2 domain, four WW domains that interact with PY motifs, and a catalytic HECT domain. HSV UL56 (HSV-1, 234 aa; HSV-2, 235 aa) contains three PY motifs and a predicted transmembrane domain (TMD). (B) Infection with wild-type (HSV-1; F, HSV-2; 186) and various mutant HSV (HSV-1; US3-deletion mutant R7041, UL13 deletion mutant R7356, γ_1_34.5-deletion mutant R3616, HSV-2; US3-deletion mutant L1BR1, UL56-reverted virus ΔUL56Zrev), but not infection with UL56-deficient HSV (HSV-1; HF10, HSV-2; ΔUL56Z), caused a marked decrease of Itch. Vero cells were mock-infected or infected with wild-type or mutant viruses and harvested at 24 hpi. Nedd4 changed only in cells infected with HSV-2 viruses except ΔUL56Z. WWP2, another Nedd4-family ubiquitin ligase, showed no remarkable change. (C-D) Itch decreases as HSV-2 infection proceeds in Vero, HEp-2 (C), and HaCaT (C, D) cells. Wild-type (186) and ΔUL56Zrev infection caused a marked decrease of Itch. In ΔUL56Z-infected cells, Itch was maintained at almost constant levels for up to 12 hpi. (D) VP5 and VP16 were detected similarly in cells infected with all three viruses. α-tubulin or β-actin were used as loading controls.

UL56 is a 235 amino acid (aa), carboxyl-terminal anchored, type II membrane protein that is predicted to be inserted into the viral envelope so that the amino-terminal domain is located in the virion tegument [21]. In this topology, UL56 is predicted to have a 216 aa cytoplasmic domain containing three PPXY (PY) motifs, which are important for its interaction with Nedd4 E3 ligase (Fig. 1A).

In a previous study, Itch, a Nedd4-family ligase, was identified as a UL56-interacting protein by a yeast two-hybrid screen [11]. Itch is widely expressed in mammalian tissues, and Itch-deficient mice develop a systemic and progressive autoimmune disease that proves lethal beginning at 6 months of age [22]. Itch is composed of 862 aa with a domain architecture similar to other Nedd4-family ligases: an amino-terminal C2 domain; four protein-protein interacting WW domains, which most commonly recognize PY motifs of binding proteins; and a carboxyl terminal catalytic HECT domain (Fig. 1A). Itch targets numerous proteins and has been implicated in signal transduction, endocytosis, differentiation, and transcription [23,24].

The catalytic activities of Nedd4-family ligases are in part regulated by some PY-motif containing membrane proteins such as Nedd4-family-interacting protein-1 (Ndfip1), -2 (Ndfip2), and Nedd4-binding partner 1 (N4BP1) [25], although the mechanisms regulating the catalytic activity of Nedd4-family ligases have not been clearly defined. Ndfip proteins function as regulatory proteins of multiple Nedd4-family ligases, including Itch and Nedd4, by recruiting ligases to substrates and controlling ligase activity [26].

In this study, to elucidate the biological function of UL56 we studied the kinetics of Itch expression in HSV-2-infected cells, and also assessed the similarity between UL56 and Ndfip proteins.

## Methods

### Cells and viruses

Vero cells (African green monkey kidney cells) and HEp2 cells (human laryngeal carcinoma cell line) were obtained and maintained as previously described [12]. HaCaT cells (human keratinocyte cell line) [27] were kindly provided by Dr. Norbert E Fusenig (German Cancer Research Center, Heidelberg, Germany). HaCaT cells were maintained in Dulbecco's modified Eagle's medium supplemented with 10% fetal calf serum, 100 U/ml penicillin and 100 μg/ml streptomycin. Cell lines constitutively expressing GFP-UL56 (Vero-GFP-UL56) or GFP (Vero-GFP) were constructed as previously described [28]. Briefly, Vero cells were transfected with pEGFP-UL56 or pEGFP-N3 (Clontech, Mountain View, CA) and selected with G418 (SIGMA, St. Louis, MO). The expression of GFP-UL56 or GFP was verified with Western blot analysis and Immunofluorescence confocal microscopy. Vero-GFP-UL56 and Vero-GFP were maintained in Eagle's minimum essential medium (MEM) supplemented with 8% calf serum (CS), 100 U/ml penicillin, 100 μg/ml streptomycin, and 350 μg/ml G418. The wild-type HSV-2 strain (186) was used as the prototype strain in this study. The generation of the UL56-deletion mutant virus (ΔUL56Z) [18], the UL56-reverted virus based on ΔUL56Z (ΔUL56Zrev) [11], and the US3-deletion mutant virus (L1BR1) [29] was previously described in detail. The HSV-1 wild type strain F, the US3-deletion mutant (R7041), the UL13-deletion mutant (R7356), and the γ_1_34.5-deletion mutant (R3616) viruses were generously provided by Dr. Bernard Roizman. HSV-1 mutant HF10 [30], lacking the functional expression of UL43, UL49.5, UL55 and UL56, and latency-associated transcripts [31] was also used. Viruses were propagated and the titers of viral stocks were determined as previously described [12].

### Antibodies and reagents

The following antibodies were used: polyclonal anti-WWP2 (Abcam, Cambridge, UK), anti-Nedd4 (Millipore, Billerica, MA), anti-GFP (MBL, Nagoya, Japan) and anti-c-Myc (Santa Cruz Biotechnology, Santa Cruz, CA); monoclonal anti-VP5 (Abcam), anti-Itch (BD Transduction Laboratories, Franklin Lakes, NJ), anti-β-actin, anti-α-tubulin (SIGMA), and anti-c-Myc (Santa Cruz Biotechnology); horseradish peroxidase-conjugated goat anti-rabbit and anti-mouse IgG (Invitrogen), and Alexa Fluor 488-conjugated goat anti-rabbit and 594-conjugated goat anti-mouse IgG (Invitrogen). Protein G affinity-purified normal mouse IgG was purchased from Millipore. Polyclonal anti-UL56 [21] and anti-VP16 [32] antisera were described previously. Reagents were purchased from the following suppliers: cycloheximide (CHX) and chloroquine (CQ), SIGMA; MG132, BIOMOL International (Plymouth Meeting, PA).

### Expression vectors

*Itch *(GenBank: NM_031483), *Ndfip1 *(GenBank: NM_030571) and *Ndfip2 *(GenBank: NM_019080) cDNA were obtained from HEp-2 cells and cloned into plasmids to generate pcDNA-Itch, pMyc-Itch, pNdfip1-EGFP, and pNdfip2-EGFP. Total RNA was extracted using ISOGEN (NIPPON GENE, Tokyo, Japan), and then first-strand cDNA was synthesized by polymerase chain reaction with reverse transcription (RT-PCR) using Transcriptor First Strand cDNA synthesis Kit (Roche Applied Science, Mannheim, Germany) in accordance with the manufacturer's instructions. Fragments of *Itch*, *Ndfip1 *or *Ndfip2 *cDNAs were amplified by PCR with KOD FX (TOYOBO, Osaka, Japan) and cloned into pcDNA3.1(+) (Invitrogen), pCMV-Myc, or pEGFP-N3 (Clontech). To generate pMyc-ICP0, HSV-2 *ICP0 *cDNA (GenBank: NC_001798) was reverse transcribed and amplified from total RNA from 186-infected Vero cells (multiplicity of infection (MOI) 3 PFU/ml, 6 h post-infection) using the same procedures described above, and then cloned into pCMV-Myc. To generate pcDNA-VP5, the HSV-2 *VP5 *ORF (GenBank: NC_001798) was amplified from HSV DNA which was extracted from 186-infected Vero cells using QIAamp DNA Blood Mini Kit (QIAgen, Hilden, Germany), and cloned into pcDNA-3.1(+). pcDNA-UL56 [21] and pcDNA-UL48 (pcDNA-VP16) [32] were generated as described previously. The *UL56 *ORF was amplified by PCR from pcDNA-UL56 and cloned into pEGFP-N3 to generate pEGFP-UL56.

### Transfection and infection

Cells plated in 35-mm dishes were transfected or infected as previously described [12]. Briefly, cells were transfected with 1 μg of each plasmid using Lipofectamine 2000 (Invitrogen), and in some experiments, further infected with HSV-2 at 48 h post-transfection. Infections were routinely performed at an MOI of 3 PFU/cell (except where otherwise indicated).

### Immunoblot assay

Cell lysates were extracted and analyzed as previously described [11].

### Co-immunoprecipitation assay

In assays on infected cells, Vero cells were pre-cultured with CQ (100 μM) for 12 h, then infected with 186 and harvested at 9 h post-infection. In assays on Vero-GFP-UL56 or Vero-GFP, cells were cultured with CQ (100 μM) for 24 h and harvested. Harvested cells were lysed and clarified by centrifugation [11]. The lysates were incubated for 1 h at 4°C with the Protein G Dynabeads (Invitrogen) which were pre-incubated with anti-Itch antibody or normal mouse IgG according to the manufacturer's instructions. After washing with lysis buffer (10 mM Tris-HCl, pH 7.4, 150 mM NaCl, 1% Nonident P-40, 1 mM EDTA, 10 mM NaF, Protease Inhibitor Cocktail [SIGMA]), the immunoprecipitated proteins were eluted in 2x SDS sample buffer and subjected to Western blot analysis.

### Immunofluorescence confocal microscopy

Indirect immunofluorescence confocal microscopy was performed as previously described [12]. In brief, cells grown on cover slips were fixed in 4% paraformaldehyde for 15 min, permeabilized with 0.1% Triton X-100 for 5 min, and incubated for 1 h at room temperature sequentially with 20% normal goat serum (DAKO, Glostrup, Denmark), primary and secondary antibodies. Confocal images were captured using the Zeiss LSM510 system (Carl Zeiss, Oberkochen, Germany).

### RNA interference

siRNAs for human *Itch *(ON-TARGETplus SMARTpool L-007196-00, siItch), and a non-targeting control pool of siRNA (ON-TARGETplus Non-targeting Pool D-001810-10, siCont) were obtained from Dharmacon (Lafayette, CO). Vero cells were transfected using Lipofectamine RNAiMAX (Invitrogen) according to the manufacturer's instructions. At 48 h post-transfection, cells were used for further experiments.

### Viral replication kinetics assay

Single-step and multi-step growth experiments were performed using Vero cells as previously described [11]. Cells were treated with siRNA for 48 h, and subsequently infected with the indicated viruses at an MOI of 3 (single-step) or 0.003 (multiple-step) PFU/cell.

## Results

### HSV infection causes a marked decrease of Itch in the presence of UL56

Initially, we investigated the kinetics of Itch expression after HSV infection. Itch was markedly decreased in Vero cells infected with wild-type and various mutant HSV that expressed UL56, but not UL56-deficient mutants (HSV-1, HF10; HSV-2, ΔUL56Z) at 24 h post-infection (Fig. 1B). Itch showed no decrease in cells infected with HF10, and remained at detectable level in cells infected with ΔUL56Z. Nedd4 was detected in two forms with different electrophoretic mobilities and had decreased levels in cells infected with HSV-2 viruses except ΔUL56Z, as previously reported [11]. In contrast, WWP2, another Nedd4-family ubiquitin ligase, which has been also identified as a UL56-interacting protein by a yeast two-hybrid screen [11], showed no remarkable change after viral infection. In time-course experiments, Itch decreased markedly as the infection with wild-type (186) or UL56-reverted (ΔUL56Zrev) viruses in multiple cell lines: Vero, HEp-2 and HaCaT cells (Fig. 1C). In ΔUL56Z-infected cells, Itch was maintained at almost constant levels for up to 12 h post-infection whereas slightly decreased at 24 h post-infection (Fig. 1C, D). Thus, HSV infection causes a decrease of Itch, and HSV-1 and -2 UL56 have a prominent role in the process. In addition, the small decrease of Itch in cells infected with ΔUL56Z suggests the presence of additional viral factors responsible for the decrease of Itch in the course of HSV-2 infection.

### UL56 causes the decrease of intrinsic Itch in the absence of other viral proteins

**Figure 2:**
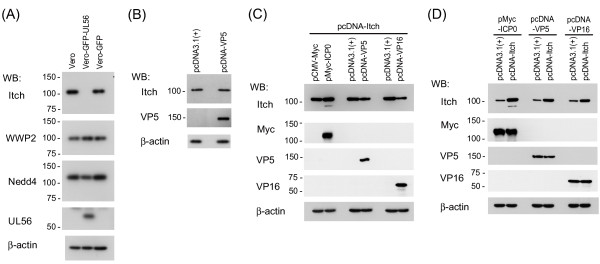
**Effects of UL56 and other viral proteins with a PY motif on Itch**. (A) Itch is markedly decreased in cells stably expressing UL56 (Vero-GFP-UL56). Lysates from Vero, Vero-GFP-UL56, or Vero-GFP cells were analyzed for Itch and other Nedd4-family ubiquitin ligases. Itch was markedly decreased in Vero-GFP-UL56 cells. (B) VP5 did not decrease endogenous Itch. Vero cells were transfected with plasmids encoding VP5 (pcDNA-VP5) or control plasmids (pcDNA3.1(+)). The levels of Itch did not change in cells transfected with pcDNA-VP5. (C) VP5 and VP16, but not ICP0, caused the decrease of exogenous Itch. Vero cells were co-transfected with plasmids encoding Itch (pcDNA-Itch) and plasmids encoding a viral protein (pMyc-ICP0, pcDNA-VP5, or pcDNA-VP16) or control plasmids (pCMV-Myc or pcDNA3.1(+)). The levels of Itch decreased in cells transfected with pcDNA-VP5 or pcDNA-VP16. (D) Overexpression of Itch has no effect on the protein levels of VP5, VP16, or ICP0. Vero cells were co-transfected with plasmids encoding a viral protein (pMyc-ICP0, pcDNA-VP5 or pcDNA-VP16) and either pcDNA-Itch or control plasmids (pcDNA-3.1(+)). The levels of viral proteins did not change with the overexpression of Itch. β-actin was used as a loading control.

We next investigated whether UL56 causes the decrease of Itch without other HSV-2 proteins using stable UL56 transfected cells (Vero-GFP-UL56). Vero-GFP-UL56 was similar in morphology and growth properties to GFP-expressing Vero cells (Vero-GFP) that were used as a control (data not shown). Itch was markedly decreased in Vero-GFP-UL56 compared to control cells (Fig. 2A). In contrast, WWP2 and Nedd4 showed relatively no decrease in Vero-GFP-UL56. These data suggest that UL56 specifically decreases Itch in the absence of any other viral proteins. We have previously reported that transient overexpression of UL56 caused the decrease of exogenous Nedd4 but had no apparent effect on endogenous Nedd4 [11]. This discrepancy may be due to the relatively low transfection efficiency (approx. 20%) in the previous study.

We further explored other possible HSV-2 proteins involved in the decrease of Itch. HSV-1 and -2 genomes encode four proteins with PY motif(s): VP5, a major capsid protein, with one motif; VP16, a tegument protein which activates the transcription of immediately early gene, with one motif; ICP0, a promiscuous transactivator, with one motif [7]; and UL56, a tegument protein with three motifs. None of the three viral proteins with one PY motif (VP5, VP16, ICP0) decreased endogenous Itch (data for VP5 is shown in Fig 2B), whereas VP5 and VP16 caused the decrease of overexpressed Itch in co-expressing cells (Fig. 2C). These results indicated that VP5 and VP16 modulate the protein level of overexpressed Itch in the absence of other viral proteins.

On the other hand, overexpression of Itch did not affect the level of VP5, VP16, or ICP0 in co-expressing cells (Fig. 2D). In infection experiments, both VP5 and VP16 were detected at the same level in cells infected with 186 and those infected with ΔUL56Z despite the substantially different level of Itch expression (Fig. 1D). These results suggest that VP5 and VP16 induce the decrease of overexpressed Itch although Itch has no apparent effect on VP5, VP16, or ICP0 levels.

### UL56 promotes lysosome- and proteasome-mediated degradation of Itch

**Figure 3:**
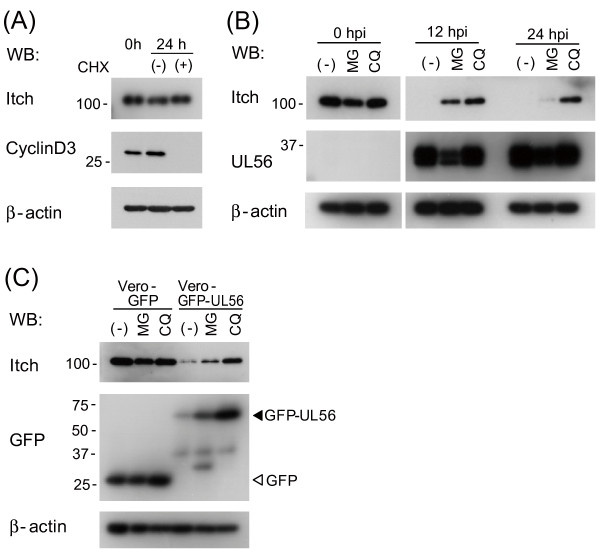
**Lysosome inhibitor and proteasome inhibitor block the decrease of Itch**. (A) Itch expression is stable in the absence of UL56. Vero cells were cultured for 24 h with or without cycloheximide (CHX, 100 μg/ml) and the lysates were analyzed for Itch expression. Cyclin D3 was used as a control. (B) Vero cells were mock-treated or treated for 12 h with either MG132 (10 μM) or chloroquine (CQ, 100 μM) and infected with wild-type HSV-2. Itch was detected in both cells treated with MG132 and those with CQ at 12 hpi, but only in those with CQ at 24 hpi. (C) The decrease of Itch is blocked by a lysosome inhibitor and partially by a proteasome inhibitor in cells stably expressing UL56. Vero, Vero-GFP-UL56, or Vero-GFP cells were mock-treated or treated with either MG132 (10 μM) or CQ (100 μM) for 24 h. β-actin was used as a loading control.

To clarify the mechanism by which the protein levels of Itch were decreased, we investigated the stability of Itch in the presence of inhibitors. The treatment of uninfected cells with the protein synthesis inhibitor cyclohexemide (CHX, 100 μg/ml) for 24 h did not alter the level of Itch (Fig. 3A), indicating that Itch is very stable in nature. In HSV-2-infected cells, the decrease of Itch was greatly blocked by chloroquine (CQ, 100 μM), a lysosome inhibitor, and only partially blocked by MG132 (10 μM), a proteasome inhibitor (Fig. 3B). Collectively, these results suggest that Itch may be degraded by the lysosome pathway and in part by the proteasome pathway in HSV-2-infected cells. The decrease of Itch in Vero-GFP-UL56 cells was also blocked by CQ and partially by MG132 (Fig. 3C). Therefore, UL56 possibly promotes the degradation of Itch via the lysosome and proteasome in HSV-2-infected cells and UL56-expressing cells. In Vero-GFP-UL56 cells, the amount of GFP-UL56 was increased significantly by the addition of MG132 and CQ, suggesting that UL 56 is degraded together with Itch in this case.

### HSV-2 UL56 interacts with Itch and changes the subcellular localization of Itch

**Figure 4:**
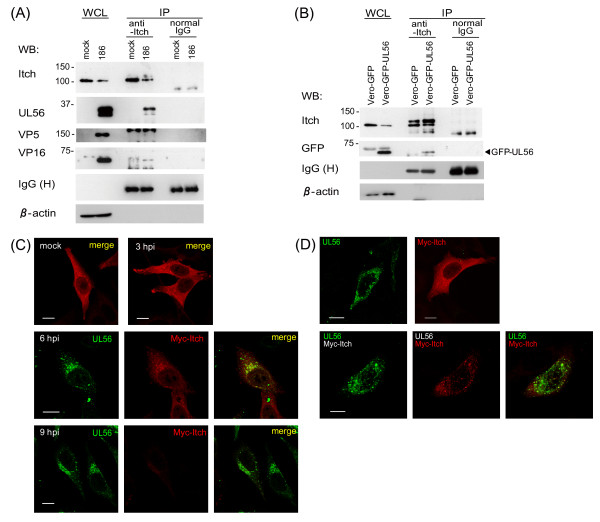
**UL56 interacts with Itch and changes the subcellular localization of Itch**. Co-immunoprecipitation assay on HSV-2-infected cells (A) or stably UL56-expressing cells (B). (A)Vero cells were treated with chloroquine (CQ) for 12 h and subsequently mock-infected or infected with wild-type HSV-2 (186). Whole cell lysates (WCL) were immunoprecipitated (IP) with an anti-Itch antibody at 9 hpi. (B) WCL from Vero-GFP or Vero-GFP-UL56 cells treated with CQ for 24 h and immunoprecipitated with an anti-Itch antibody. UL56 was detected in the Itch-immunoprecipitates in HSV-2-infected cells (A) and stably UL56-expressing cells (B). β-actin was used as a loading control. Confocal immunofluorescence analysis of the subcellular localizations of Myc-Itch and UL56 in HSV-2-infected cells (C) or co-expressing cells (D). (C) HEp-2 cells were transfected with plasmids encoding Myc-Itch and subsequently infected with wild-type HSV-2. The Myc-Itch (red) showed the altered subcellular distribution with the reduced signal intensity after 6 hpi, when UL56 (green) became detectable. Myc-Itch colocalized with UL56 in the vesicular pattern. (D) HEp-2 cells were transfected with plasmids encoding UL56 (pcDNA-UL56) and/or pCMV-Myc-Itch. In co-expressing cells (bottom panels), Myc-Itch changed its distribution and colocalized with UL56 in the vesicular pattern. The Myc signal was reduced in co-expressing cells. Scale bars, 10 μm.

We next investigated whether UL56 and Itch interact. Co-immunoprecipitation assay using anti-Itch antibody revealed that UL56 was associated with Itch in cells infected with wild-type HSV-2 (Fig. 4A) and cells stably expressing UL56 (Fig. 4B). These results indicate that UL56 interacts with Itch during HSV-2 infection and that no other viral proteins are required for the interaction. In infected cells, VP16 was also detected at a much lower level whereas VP5 was not detected (Fig. 4A).

Confocal immunofluorescence analysis revealed the colocalization of UL56 and Itch in HSV-2-infected cells and transiently UL56-expressing cells. Myc-tagged Itch (Myc-Itch) was mainly distributed throughout the cytoplasm with partial vesicular distribution in uninfected cells (Fig. 4C, top left panel). The Myc-Itch showed reduced signal intensity and altered subcellular distribution after 6 hpi, concomitant with UL56 detection (Fig. 4C, middle and bottom panels). Myc-Itch accumulated in the perinuclear region with punctate distribution and colocalized with UL56 at 6 hpi and 9 hpi. These results support the view that Itch interacts with UL56, and decreases during HSV-2 infection. Co-expression with UL56 also reduced signal intensity and altered the distribution of Myc-Itch (Fig. 4D); Myc-Itch showed the clear vesicular distribution and colocalized with UL56. These results highlight that UL56 interacts directly with Itch and causes Itch to decrease even without other viral proteins.

### siRNA knockdown of Itch has no apparent effect on the growth of either wild-type or UL56-deficient HSV-2

**Figure 5:**
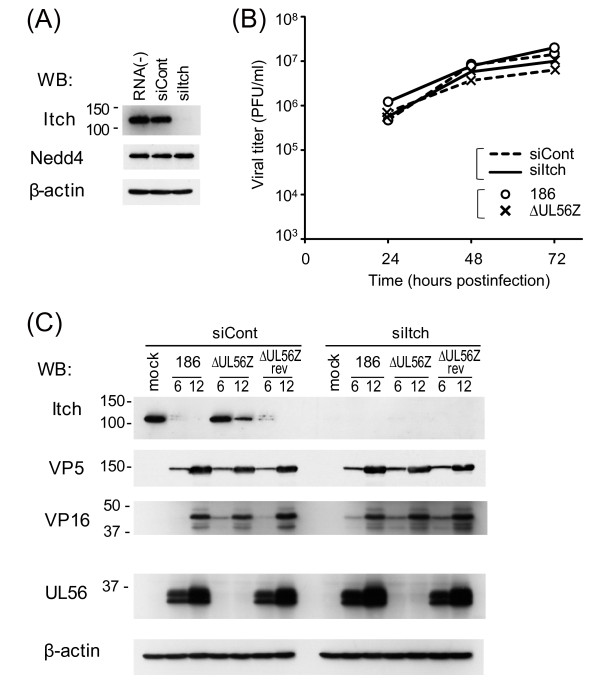
**siRNA knockdown of Itch has no apparent effect on the viral growth**. Vero cells were mock-transfected (RNA [-]) or transfected with either negative control siRNAs (siCont) or siRNAs specific to Itch (siItch) for 48 h (A), and subsequently infected with indicated viruses (B, C). (A) siItch efficiently and specifically down-regulates protein levels of Itch, whereas levels of Nedd4 and β-actin were constant. (B) A multi-step growth curve in siRNA-treated cells (MOI 0.003 PFU/ml). Both wild-type (186) and ΔUL56Z viruses showed similar growth kinetics in siCont-treated cells and siItch-treated cells. (C) Expression of Itch and viral proteins with PY motifs (VP5, VP16, and UL56) by immunoblot. There was no difference in viral protein level between siCont- and siItch-treated cells. β-actin was used as a loading control.

To assess the role of Itch in HSV-2 replication, the effect of Itch knockdown on the efficiency of viral growth in Vero cells was measured. Itch protein levels were efficiently and specifically down-regulated by Itch siRNA (siItch) (Fig. 5A). Wild-type viruses showed similar growth kinetics in siCont-treated cells and siItch-treated cells both in multiple- (MOI 0.003, Fig. 5B) and single- (MOI 3, data not shown) growth experiments. ΔUL56Z also showed similar growth kinetics in siCont- and siItch-treated cells (Fig. 5B). Additionally, a major capsid protein VP5 and tegument proteins, VP16 and UL56, showed similar expression patterns in siCont- and siItch-treated cells (Fig. 5C). Thus, the knockdown of Itch did not influence the replication of wild-type and UL56-deficient HSV-2 in Vero cells.

### HSV-2 UL56 colocalizes with Ndfip proteins

**Figure 6:**
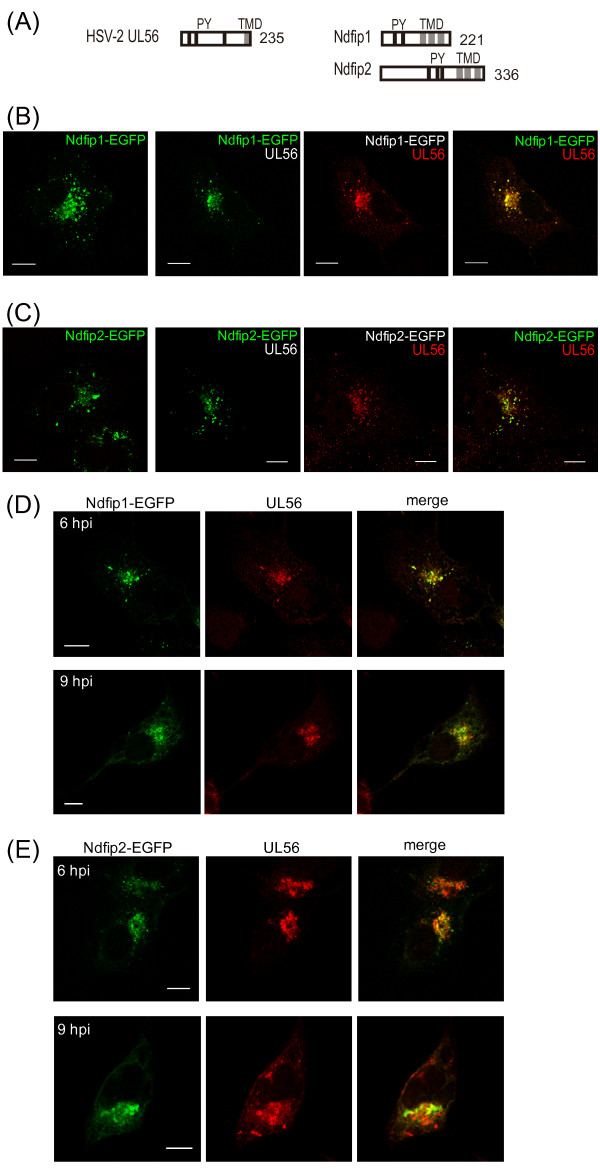
**UL56 co-localizes with Ndfip1 and Ndfip2**. (A) Schematic representation of UL56 and Ndfip proteins. HSV-2 UL56 (235 aa) contains three PY motifs and one predicted transmembrane domain (TMD). Ndfip1 (221 aa) and Ndfip2 (336 aa) contain two and three PY motifs respectively, and three TMD. (B-C) HEp-2 cells were transfected with plasmids encoding either Ndfip1-EGFP (pNdfip1-EGFP) (B) or Ndfip2-EGFP (pNdfip2-EGFP) (C) alone (left panels), or in combination with plasmids encoding UL56 (right panels). UL56 colocalized with Ndfip1-EGFP and Ndfip2-EGFP in co-expressing cells. (D-E) HEp-2 cells were transfected with pNdfip1-EGFP (D) or pNdfip2-EGFP (E), and subsequently infected with wild-type HSV-2. UL56 partially colocalized with Ndfip1-EGFP and Ndfip2-EGFP in infected cells. Scale bars, 10 μm.

Ndfip1 and Ndfip2 are small membrane proteins with multiple PY motifs (Fig. 6A) that regulate Nedd4 family ligases including Itch and Nedd4 by directly controlling ligase activity and relocating ligases [26]. To provide evidence of similarity between UL56 and Ndfip proteins, we investigated whether UL56 and Ndfip proteins colocalize in HSV-2-infected cells and cells co-expressing UL56 and a Ndfip protein. EGFP-tagged-Ndfip1 (Ndfip1-EGFP) (Fig. 6B) and -Ndfip2 (Ndfip2-EGFP) (Fig. 6C) showed vesicular distribution with accumulation to the perinuclear space, consistent with results of other studies [33,34]. When co-expressed with UL56, Ndfip1-EGFP and Ndifip2-EGFP did not change their subcellular distribution and they largely colocalized with UL56. In HSV-2 infected cells, Ndfip proteins altered their distribution and formed perinuclear clumps (Fig. 6D, E). UL56 showed a similar distribution pattern and accumulated in the perinuclear space, but only partially colocalized with Ndfip proteins.

## Discussion

This study demonstrates that HSV-2 UL56 interacts with the Nedd4-family ubiquitin ligase Itch, and moreover, targets Itch for degradation primarily via the lysosome pathway and partially via the ubiquitin-proteasome pathway in the course of HSV-2 infection. UL56 interacted with Itch and induced the degradation of Itch independent of any other viral proteins. To the best of our knowledge, this is the first report demonstrating that an HSV protein interacts with Itch. In addition, UL56 and Ndfip proteins, regulatory proteins of Nedd4 and Itch, showed similar subcellular distribution and colocalized.

Itch differed from Nedd4, another UL56-interacting E3 ligase, in the following aspects: Itch decreased in both HSV-1-infected cells and HSV-2-infected cells, whereas Nedd4 decreased only in HSV-2-infected cells; endogenous Itch was degraded much more efficiently than Nedd4 in UL56-expressing cells; Itch was degraded primarily by the lysosome pathway whereas Nedd4 was degraded by the proteasome pathway [11]; and Itch was co-immunoprecipitated with a major tegument protein VP16 whereas Nedd4 was not [11]. UL56 caused more striking changes in Itch than in Nedd4 as a whole. The distinct effects of UL56 on Itch and Nedd4 support the view that each of Nedd4-family ligases is regulated in the specific way in spite of sharing many common properties [35]. HSV-1 UL56 (234 aa) and HSV-2 UL56 (235 aa) share three PY motifs and one C-terminal transmembrane domain, and exhibit 62.6% identity on the amino acid level. UL56 itself and/or other viral proteins could account for the different effect of HSV-1 and HSV-2 on Nedd4 and Itch.

To our knowledge, UL56 is the first example of a protein which induces Itch to degrade except Itch itself. Itch is regulated by multiple mechanisms: phosphorylation mediated by Jun amino-terminal kinase [36] and Fyn [37]; conformational change and relocation induced by adaptor/regulatory proteins (Ndfip-1 [38] and -2 [26], and N4BP1 [25]); and modulation of the level of Itch ubiquitination mediated by Itch itself [36], FAM/USP9X [39], and Akt1 [40]. Little has been done to clarify how Itch degradation is controlled because of its high stability, and moreover, the limited results obtained so far are controversial. One report showed that autoubiquitinated Itch is degraded by the proteasome [39], however others showed Itch is very stable even in polyubiquitinated state, and the level of ubiquitination has no discernible impact on Itch stability [40,41]. UL56 originally induced Itch to degrade via primarily the lysosome and partially the proteasome pathways. From the view point of the degradation of ubiquitinated proteins, this result concurs with the report which showed ubiquitinated proteins can undergo lysosomal degradation [42]. In addition, a proteasome inhibitor blocked the degradation at 12 hpi but not at 24 hpi, whereas a lysosome inhibitor blocked both at 12 and 24 hpi. These data suggest that the degradation pathway could change during the course of HSV-2 infection.

Of the three viral proteins with PY motif(s) other than UL56, only VP16 was detected in the Itch-immunoprecipitates, albeit at a much lower level than UL56. VP16 is a tegument protein which activates viral transcription of immediate early genes after infection and plays an essential role during assembly in the late phase of infection [43]. VP16 interacts with multiple envelope- and tegument- proteins including UL36 (VP1/2), and appears to function in linking the outer tegument/glycoprotein and capsid/inner tegument complexes [44,45]. UL36 is a large inner tegument protein with the deubiquitinating activity [10], and required for the addition of VP16 to the viral capsid [46]. It is noteworthy that VP16 associates with Itch, a cellular E3 ligase, and also with UL36, a viral DUB.

We explored the possibility that additional viral proteins mediated the decrease of Itch, since there was a small decrease in cells infected with UL56-deficient HSV-2. Two of three HSV-2 proteins with one PY motif other than UL56, VP5 and VP16 caused the decrease of overexpressed Itch in co-expressing cells. In contrast, ICP0, a viral component with promiscuous transactivity and ubiquitin ligase activity, did not influence Itch expression. These results indicate that only specific viral proteins with PY motifs are capable of inducing Itch degradation. In addition, transient expressions of VP5 and VP16 caused no decrease of endogenous Itch, whereas stable expression of UL56 caused the striking decrease of endogenous Itch. These results support the notion that UL56 plays a prime role, and VP5 and VP16 can play secondary roles in the decrease of Itch during HSV-2 infection. The reason why UL56-deficient HSV-1 did not cause the decrease of Itch remains unknown.

The experiments with inhibitors provided insights into the mechanism of UL56 degradation. Treatment with a lysosome inhibitor caused the increase of UL56 in cells stably expressing UL56. This result suggests that UL56 is also degraded via the lysosome pathway in UL56-expressing cells. Interestingly, treatment with a proteasome inhibitor also caused the increase of UL56, although the effect was minimal, suggesting that the proteasome pathway is also involved in UL56 degradation. Given that UL56 is lysine-free and not ubiquitinated [11], some additional factors may be involved in the degradation of UL56. In this study, we used only one lysosome-inhibitor and one-proteasome-inhibitor, and did not analyze ubiquitinated substrates or free ubiquitin. Further investigation is needed concerning the turnover of UL56.

We also investigated whether Itch can change the protein levels of VP5, VP16, and ICP0 because they contain a PY motif and also lysine residues, which are targets of ubiquitination. Contrary to expectations, overexpression of Itch did not affect the level of VP5, VP16, or ICP0, and knockdown of Itch did not change the expression patterns of VP5 and VP16 during the course of infection. Thus, the levels of these three viral proteins do not appear to be regulated by Itch.

siRNA knockdown of Itch has no apparent effect on the growth of either wild-type or UL56-deficient HSV-2 in Vero cells. Itch is reported to be involved in viral replication and pathogenicity in Epstein-Barr virus (EBV), which belongs to Gammaherpesvirinae family, and Moloney murine leukemia virus (MoMLV). Itch interacts with latent membrane protein (LMP) 2A of EBV and down-regulates LMP2A activity in B-cell signaling [47,48], and rescues a release-deficient MoMLV independent of PY motif of the Gag protein [49]. More investigations are needed to elucidate how the decrease of Itch is involved in the replication and pathogenicity of HSV-2.

UL56 and Ndfip proteins share some common features: small membrane proteins (Ndfip1, 221 aa; Ndfip2, 336 aa; and UL56, 234 aa); contain multiple PY motifs in the cytoplasmic domains (Ndfip1, two; Ndfip2 and UL56, three); interaction with Nedd4 and Itch via PY motifs; and relocate Nedd4 and Itch for degradation. This study revealed that UL56 and Ndfip proteins are similar in their subcellular localization. In co-expressing cells, UL56 and Ndfip proteins colocalized to the vesicles. UL56 localized primarily the TGN and early endosomes [12], while Ndfip proteins localized to the TGN, early endosomes, and late endosomes/multi vesicular bodies [33,34]. The partial colocalization of UL56 and either Ndfip-1 or -2 in infected cells suggests that UL56 and Ndfip proteins behave similarly during the course of HSV infection. It is interesting that the viral protein UL56 shares so many properties with cellular regulatory proteins of Itch and Nedd4.

## Conclusions

This study demonstrates that HSV-2 UL56 interacts with a Nedd4-family ubiquitin ligase Itch, and moreover, relocates Itch and induces Itch to degrade in the course of HSV-2 infection. UL56 caused more striking changes in Itch than in Nedd4 as a whole. In addition, UL56 shared multiple common properties with Ndfip proteins. In light of these results, we propose that UL56 functions as a regulatory protein of Itch. The mechanism, function and significance of regulating Itch in HSV-2 infection remain unclear and warrant further investigation.

## Competing interests

The authors declare that they have no competing interests.

## Authors' contributions

YU and YN designed the research, YU, CL, and MK performed the experimental work, YU conducted the data analysis and drafted the manuscript, and FG, HK, and YN participated in the data analysis and review of the manuscript. All authors read and approved the final manuscript.
